# *Dnmt3b* Deficiency in Adipocyte Progenitor Cells Ameliorates Obesity in Female Mice

**DOI:** 10.3390/ijms27020861

**Published:** 2026-01-15

**Authors:** Yifei Huang, Sean Yu, Qiang Cao, Weiqing Tang, Jia Jing, Bingzhong Xue, Hang Shi

**Affiliations:** 1 Department of Biology, Georgia State University, Atlanta, GA 30303, USA; yhuang62@student.gsu.edu (Y.H.); caoqiang78@gmail.com (Q.C.); jjing@gsu.edu (J.J.); 2 Centennial High School, Ellicott City, MD 21042, USA; seanyu941@gmail.com; 3 Division of Endocrinology, Diabetes and Nutrition, Department of Medicine, University of Maryland School of Medicine, Baltimore, MD 21201, USA; weiqing.tang@som.umaryland.edu

**Keywords:** *Dnmt3b*, DNA methylation, obesity

## Abstract

Obesity arises from chronic energy imbalance, where energy intake exceeds energy expenditure. Emerging evidence supports a key role of DNA methylation in the regulation of adipose tissue development and metabolism. We have recently discovered a key role of DNA methylation, catalyzed by DNA methyltransferase 1 or 3a (*Dnmt1* or *3a*), in the regulation of adipocyte differentiation and metabolism. Here, we aimed to investigate the role of adipose progenitor cell *Dnmt3b*—an enzyme mediating de novo DNA methylation—in energy metabolism and obesity. We generated a genetic model with *Dnmt3b* knockout in adipocyte progenitor cells (PD3bKO) by crossing *Dnmt3b* floxed mice with Platelet-derived growth factor receptor alpha (PDGFRα) Cre mice. *Dnmt3b* deletion in adipocyte progenitors enhanced thermogenic gene expression in brown adipose tissue, increased overall energy expenditure, and mitigated high-fat diet (HFD)-induced obesity in female mice. PD3bKO mice also displayed a lower respiratory exchange ratio (RER), indicative of a metabolic shift favoring fat utilization as an energy source. Furthermore, female PD3bKO mice exhibited improved insulin sensitivity alongside their lean phenotype. In contrast, male PD3bKO mice showed no changes in body weight but demonstrated decreased insulin sensitivity, revealing a sexually dimorphic metabolic response to *Dnmt3b* deletion in adipose progenitor cells. These findings underscore the critical role of *Dnmt3b* in regulating energy homeostasis, body weight, and metabolic health, with significant implications for understanding sex-specific mechanisms of obesity and metabolism.

## 1. Introduction

Obesity is closely associated with a panel of metabolic disorders such as type 2 diabetes, hypertension, dyslipidemia, and cardiovascular diseases [1]. Obesity arises from a chronic energy influx due to energy intake over expenditure [1]. Thus, a better understanding of the mechanism governing energy metabolism may provide a therapeutic strategy for the treatment of obesity and related metabolic diseases.

Adipose tissue plays a central role in regulating energy homeostasis by balancing energy storage, release, and dissipation. This dynamic process is mediated by three distinct types of adipocytes: white, brown, and beige. White adipose tissue (WAT) stores excess energy as triglycerides through hypertrophy, hyperplasia, or both, and releases fatty acids via lipolysis to fuel other organs during energy demand [2]. In contrast, brown adipose tissue (BAT) dissipates energy through adaptive thermogenesis, employing both UCP1-dependent and independent mechanisms [3,4,5,6]. Beige adipocytes, sporadically dispersed in WAT depots, are primarily induced by β-adrenergic stimulation triggered by cold exposure or β-adrenergic agonists [7]. Sharing morphological and biochemical features with BAT, beige adipocytes also contribute to thermogenesis [7]. Together, these adipocyte types perform distinct yet complementary roles in maintaining energy homeostasis.

Obesity, like many other complex diseases, arises from the interplay between genetic and environmental factors, such as diet. One mechanism by which environmental factors influence gene expression is through epigenomic reprogramming. Epigenetic regulation thus serves as a critical molecular link between obesity and environmental factors [8,9]. DNA methylation—a common epigenetic mechanism—involves the covalent addition of a methyl group to cytosine, often at CpG sites. This modification typically occurs in gene promoters and 5′ regions, where CpG sites are enriched [10,11]. Hypomethylation in promoter regions generally activates gene transcription, while hypermethylation silences genes by disrupting the binding of transcriptional activators or by cooperating with histone modifications to alter DNA accessibility [11,12]. Three functional DNA methyltransferases (DNMTs)—namely, DNMT1, DNMT3A, and DNMT3B—catalyze DNA methylation in distinct contexts [11]. DNMT1, which prefers hemimethylated DNA, primarily maintains DNA methylation patterns during replication. In contrast, DNMT3A and DNMT3B are responsible for de novo methylation, establishing new methylation patterns [11]. However, emerging evidence suggests that DNMT1 may also contribute to de novo methylation under certain circumstances [13].

We recently identified DNA methylation as a key regulator of adipocyte development and metabolism. Specifically, we discovered that DNA methylation, catalyzed by DNMT1 and DNMT3A, has a biphasic role in 3T3-L1 differentiation: promoting adipogenesis during the early stages while inhibiting lipogenesis at later stages [14]. Moreover, we found that DNMT1, DNMT3A and DNMT3B in brown fat are critical for regulating thermogenesis and diet-induced obesity in mice [15]. We aimed to investigate the role of DNMT3B in adipose progenitor cells and its impact on energy metabolism in adipose tissue. To achieve this, we generated a genetic model with *Dnmt3b* knockout in adipocyte progenitor cells (PD3bKO) by crossing *Dnmt3b* floxed mice with Platelet-derived Growth Factor Receptor Alpha (PDGFRα) Cre mice. We then characterized the metabolic phenotype of these mice under a high-fat diet (HFD).

## 2. Results

### Dnmt3b Deficiency in Adipose Progenitor Cells Ameliorates Diet-Induced Obesity in Female Mice

**Figure 1:**
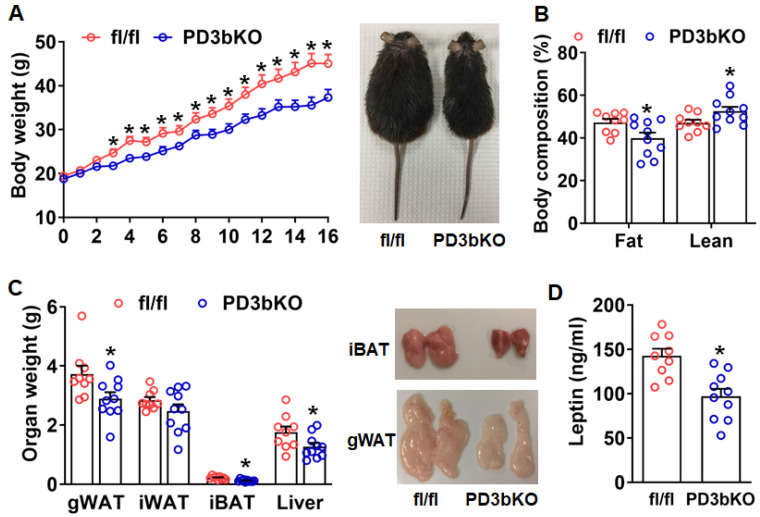
*Dnmt3b* deficiency in adipose progenitor cells prevents HFD-induced obesity in female mice. Six-week old female PD3bKO and their littermate control fl/fl mice were put on a HFD for 16 weeks. (**A**) Body weight growth curve in female PD3bKO and fl/fl mice. (**B**) Body composition measured by a Bruker NMR body composition analyzer in female PD3bKO and fl/fl mice. (**C**) Organ weight of gonadal WAT (gWAT), inguinal white adipose tissue (iWAT), interscapular brown adipose tissue (iBAT), and liver in female PD3bKO and fl/fl mice. (**D**) Circulating leptin levels in female PD3bKO and fl/fl mice. All data are expressed as mean ± SEM; *n* = 9–10/group; * *p* < 0.05 vs. fl/fl.

In this study, we generated a genetic model with *Dnmt3b* knockout in adipocyte progenitor cells (PD3bKO) by crossing *Dnmt3b* floxed mice with Platelet-Derived Growth Factor Receptor Alpha (PDGFRα) Cre mice. PDGFRα is a key marker of adipocyte progenitor cells that regulates adipogenesis in vivo [16,17]. The PDGFRα Cre line has been widely utilized for adipocyte lineage tracing and progenitor cell adipogenesis studies [16,17,18,19,20]. Deletion of *Dnmt3b* in adipocyte progenitor cells resulted in a marked reduction in *Dnmt3b* mRNA in inguinal white adipose tissue (iWAT), interscapular brown adipose tissue (iBAT), and gonadal WAT (gWAT, or epidydimal WAT: eWAT for male) of both female and male PD3bKO mice (Supplementary Materials Figure S1A,B). In contrast, *Dnmt1* and *Dnmt3a* expression remained largely unchanged across these fat depots in both female and male PD3bKO mice, with the exception of a modest decrease in *Dnmt3a* in iBAT of male PD3bKO mice (Supplementary Materials Figure S1C–F). To investigate the role of *Dnmt3b* in diet-induced obesity, we fed PD3bKO mice with a high-fat diet (HFD) and characterized their metabolic phenotype. Female PD3bKO mice displayed significantly lower body weight compared to their littermate fl/fl controls (Figure 1A). Analysis of body composition using a Minispec NMR body composition analyzer revealed decreased body fat content and increased lean mass in female PD3bKO mice (Figure 1B), which was associated with reduced weights of interscapular BAT (iBAT), gonadal WAT (gWAT), and liver (Figure 1C). Consistent with decreased fat mass, circulating leptin levels were also reduced in PD3bKO mice (Figure 1D).

**Figure 2:**
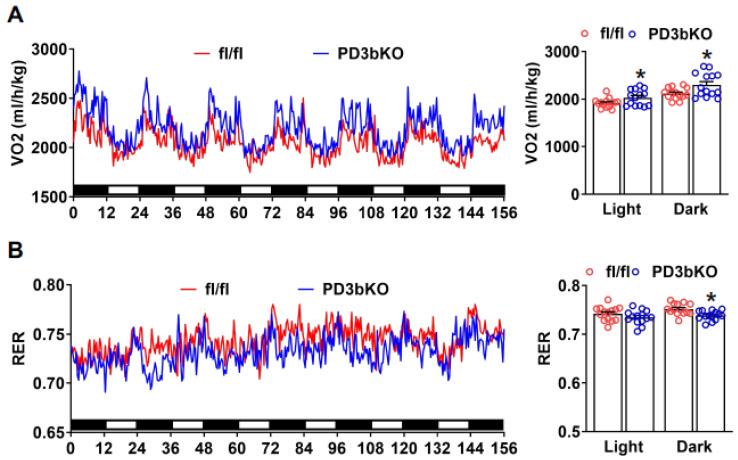
*Dnmt3b* deficiency in adipose progenitor cells promotes energy expenditure in female mice. Female PD3bKO and fl/fl mice fed HFD were put in TSE PhenoMaster metabolic cage system for metabolic characterization. (**A**) Oxygen consumption. (**B**) Respiratory exchange ratio (RER). All data are expressed as mean ± SEM; *n* = 9–10/group; * *p* < 0.05 vs. fl/fl.

**Figure 3:**
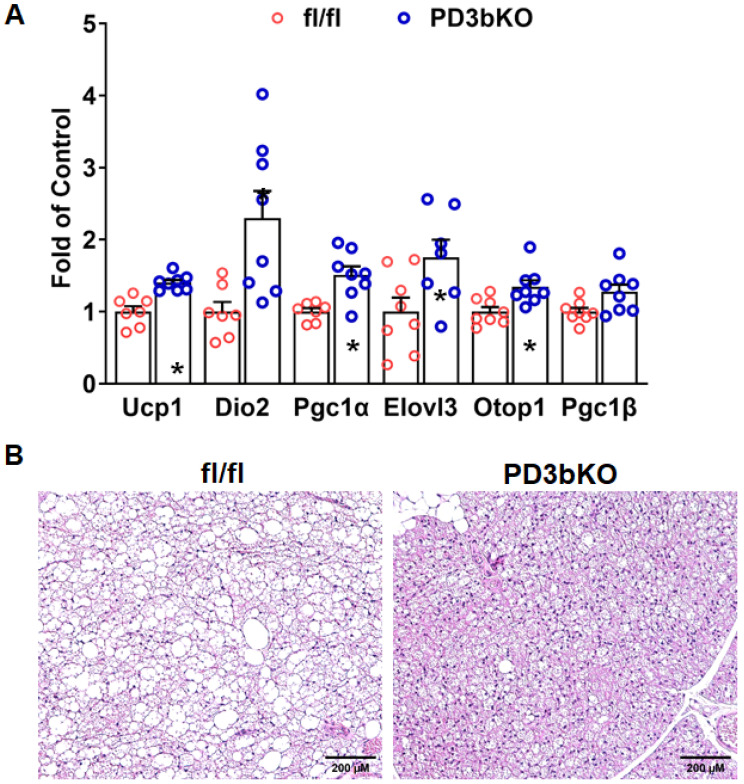
*Dnmt3b* deficiency in adipose progenitor cells promotes brown fat thermogenic program in female mice fed HFD. Six-week old female PD3bKO and their littermate control fl/fl mice were put on a HFD for 16 weeks. (**A**) Quantitative RT-PCR analysis of thermogenic gene expression in iBAT (*n* = 7–8/group). (**B**) Hematoxylin and eosin (H&E) staining of iBAT. All data are expressed as mean ± SEM; * *p* < 0.05 vs. fl/fl.

To elucidate the pathways contributing to the reduced adiposity in PD3bKO mice, we assessed energy expenditure using the PhenoMaster metabolic cage system. PD3bKO mice exhibited higher oxygen consumption (Figure 2A), indicating increased energy expenditure. Moreover, a lower respiratory exchange ratio (RER) was observed (Figure 2B), suggesting a preference for fat as the primary energy source in knockout mice. Interestingly, we also found reduced food intake in PD3bKO mice compared to floxed controls (Supplementary Materials Figure S2). Further analysis of thermogenic gene expression in iBAT using quantitative PCR revealed significant upregulation of thermogenic markers, including *Ucp1*, *Pgc1α*, *Dio2*, and *Elovl3*, in female PD3bKO mice (Figure 3A), accompanied by smaller brown adipocytes (Figure 3B). Collectively, these findings suggest that the lean phenotype observed in female PD3bKO mice results from increased energy expenditure and decreased food intake.

**Figure 4:**
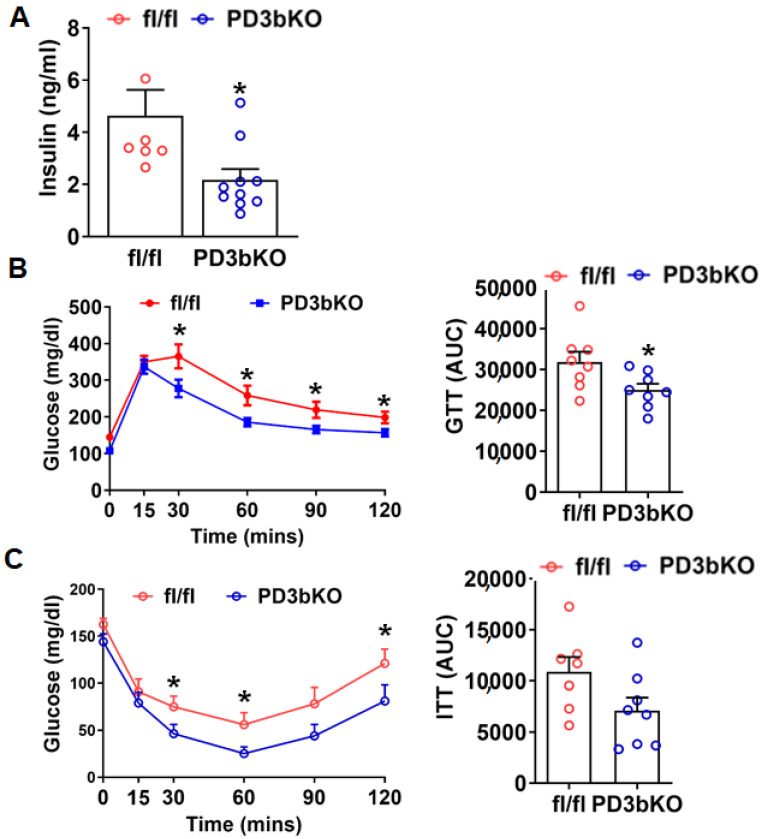
*Dnmt3b* deficiency in adipose progenitor cells improves insulin sensitivity in female mice fed HFD. Six-week old female PD3bKO and their littermate control fl/fl mice were put on a HFD for 16 weeks. (**A**) Circulating insulin levels. (**B**) Glucose tolerance test (GTT) in female PD3bKO and fl/fl mice. (**C**) Insulin tolerance test (ITT) in female PD3bKO and fl/fl mice. All data are expressed as mean ± SEM; *n* = 7–8/group; * *p* < 0.05 vs. fl/fl.

Given the close relationship between adiposity, glucose homeostasis, and insulin sensitivity, we next examined glucose metabolism in PD3bKO mice on HFD. Female PD3bKO mice exhibited lower fasting insulin levels, indicative of improved insulin sensitivity (Figure 4A). Consistent with these findings, glucose tolerance tests (GTT) and insulin tolerance tests (ITT) revealed enhanced glucose tolerance and insulin sensitivity in PD3bKO mice compared to controls (Figure 4B,C).

**Figure 5:**
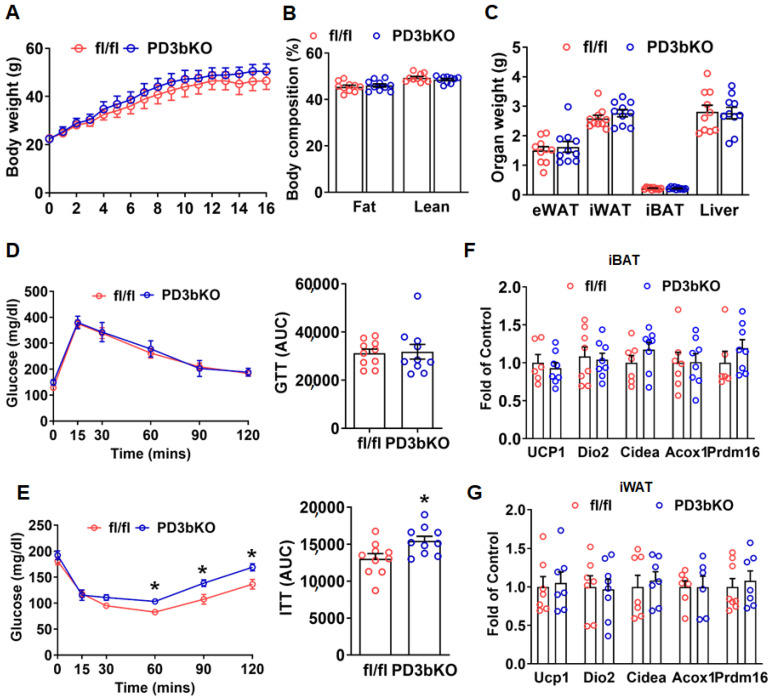
Dnmt3b deficiency in adipose progenitor cells does not change body weight in male mice fed HFD. Six-week old male PD3bKO and their littermate control fl/fl mice (*n* = 10/group) were put on a HFD for 16 weeks. (**A**) Body weight growth curve in male PD3bKO and fl/fl mice. (**B**) Body composition measured by a Bruker NMR body composition analyzer in male PD3bKO and fl/fl mice. (**C**) Organ weight of epididymal WAT (eWAT), inguinal white adipose tissue (iWAT), interscapular brown adipose tissue (iBAT), and liver in male PD3bKO and fl/fl mice. (**D**) Glucose tolerance test (GTT) in male PD3bKO and fl/fl mice. (**E**) Insulin tolerance test (ITT) in male PD3bKO and fl/fl mice. Quantitative RT-PCR analysis of thermogenic gene expression in iBAT (**F**) and iWAT (**G**) (*n* = 7/group). All data are expressed as mean ± SEM; * *p* < 0.05 vs. fl/fl.

Unlike the lean phenotype observed in female PD3bKO mice, male PD3bKO mice fed a HFD exhibited a trend toward higher body weight, though it did not reach statistical significance (Figure 5A). Moreover, male PD3bKO mice showed no significant changes in body composition or fat pad weight compared to controls (Figure 5B,C). While glucose tolerance, assessed by GTT, remained unchanged in male PD3bKO mice (Figure 5D), they displayed increased insulin resistance, as evidenced by ITT results (Figure 5E). Unlike female PD3bKO mice, male PD3bKO mice showed no change in thermogenic gene expression in iBAT and iWAT (Figure 5F,G).

**Figure 6:**
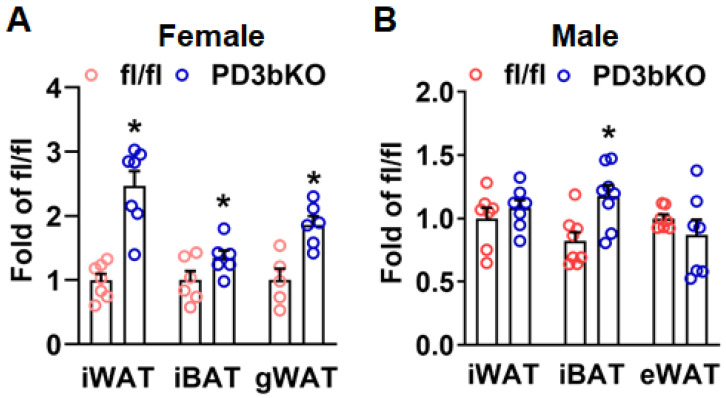
Dnmt3b deficiency in adipose progenitor cells increases *Esr1* expression in adipose tissues of female mice fed HFD. Quantitative RT-PCR analysis of *Esr1* expression in fat depots of female (**A**) and male (**B**) PD3bKO mice (*n* = 6/group). All data are expressed as mean ± SEM; * *p* < 0.05 vs. fl/fl.

Given our prior observation that DNMT1/3A-mediated methylation at the *Esr1* promoter in adipocytes suppresses estrogen receptor expression [21], we examined *Esr1* expression in adipose tissues of both male and female PD3bKO mice. We found that *Esr1* expression was increased in all fat depots of female PD3bKO mice (Figure 6A), whereas in male PD3bKO mice, *Esr1* expression was increased only in iBAT and showed a trend toward elevation in iWAT (Figure 6B).

## 3. Discussion

In this study, we demonstrated that female PD3bKO mice with *Dnmt3b* deficiency in adipocyte progenitor cells exhibit resistance to high-fat diet (HFD)-induced obesity and insulin resistance. This phenotype is associated with increased energy expenditure and reduced caloric intake. These findings align with and build upon our prior observations highlighting the significance of epigenetic regulation in obesity and metabolic disease development. The plausibility of this study was informed by prior observations. Emerging evidence has demonstrated epigenetic regulation as a key mechanism mediating the development of obesity and metabolic diseases. Obesity, a multifactorial disease, results from complex interactions between genetic and environmental factors. Environmental influences, such as diet, modulate gene expression through epigenetic reprogramming, thereby serving as a mechanistic link between external factors and disease outcomes [22,23,24,25,26]. DNA methylation, a common epigenetic modification, has been implicated in regulating genes involved in various metabolic pathways, including *Ucp1* [27], *Pgc1α* [28,29], *Pparγ* [30], *Lpl* and *aP2* [30], *leptin* [22,31], etc. Our previous work has further explored the role of DNA methylation in metabolic regulation. For instance, we demonstrated that DNA methyltransferases DNMT1 and DNMT3A exhibit stage-specific regulatory effects on 3T3-L1 adipogenesis, promoting early differentiation while inhibiting late-stage lipogenesis [14]. In addition, we also discovered that obesity-induced factors induce DNA hypermethylation at the PPARγ1 promoter via DNMT1, promoting macrophage polarization, inflammation, and the progression of insulin resistance and atherosclerosis [32]. Further, we have also shown that DNMT1/3A-mediated methylation at Esr1 promoter played an important role in regulating adipose inflammation, which may contribute to obesity-induced insulin resistance [21]. These findings motivated us to extend our focus to DNA methylation in BAT development and thermogenic function [15]. Notably, we found that *Dnmt1* or *Dnmt3a* deficiency in BAT promotes its remodeling into a skeletal myocyte-like phenotype, resulting in decreased energy expenditure and increased adiposity [15]. The present study supports a role for *Dnmt3b* in regulating thermogenic programming, potentially via epigenetic mechanisms.

In contrast to these findings, the present study reveals that female PD3bKO mice with *Dnmt3b* deletion in adipocyte progenitor cells exhibit resistance to HFD-induced obesity. Although the precise mechanisms underlying this lean phenotype remain unclear, these mice show increased thermogenic activity in interscapular BAT (iBAT). While it remains uncertain whether PDGFRα is expressed in brown adipocyte progenitor cells, which share a developmental lineage with skeletal muscle, PDGFRα has been identified in UCP1-positive beige progenitor cells [33]. PDGFRα Cre might also be expressed in BAT progenitor cells, potentially leading to Dnmt3b deletion in BAT. Consistent with this hypothesis, PD3bKO mice exhibit reduced *Dnmt3b* levels in iBAT, as shown in Supplemental Figure S1. We speculate that early *Dnmt3b* deletion in brown adipocyte progenitor cells enhances brown fat development and thermogenic function in mature brown adipocytes, thereby increasing energy expenditure and reducing adiposity. This is consistent with our recent report that deletion of *Dnmt3b* in mature brown adipocytes using Ucp-1 Cre driver ameliorates obesity in female mice [34]. These findings suggest that *Dnmt3b* may exert distinct roles in brown fat thermogenic regulation compared to *Dnmt1* and *Dnmt3a* [15]. Like many epigenetic regulators, DNMTs likely perform stage-specific functions during development. Further studies are warranted to elucidate the precise mechanisms by which *Dnmt3b* regulates adipogenesis in brown adipocyte progenitors. One limitation of the present study is the lack of direct DNA methylation measurements. Future work involving locus-specific bisulfite sequencing or methylome analysis in progenitors and mature brown adipocytes is warranted to define the precise epigenetic mechanisms.

We fully recognize that PDGFRα-Cre is not exclusively restricted to adipocyte progenitors and can be expressed in other non-adipose tissues depending on developmental context. Indeed, female PD3bKO mice exhibit reduced food intake while maintaining physical activity, both of which contribute to their lean phenotype. It remains to be determined whether these effects stem from peripheral factors, such as fat-derived secretory molecules, or whether they involve potential *Dnmt3b deletion* in neuronal progenitor cells due to PDGFRα Cre expression in the central nervous system. Our current interpretation of the data is that the primary driver of the observed metabolic changes is adipose tissue, based on the strong and consistent adipose phenotypes and thermogenic gene changes. Future studies using additional Cre drivers (e.g., more restricted adipocyte-lineage Cre lines) will be needed to determine adipose-intrinsic effects from potential contributions of other PDGFRα-expressing lineages.

Unlike female PD3bKO mice, male PD3bKO mice displayed a trend toward higher body weight and greater insulin resistance when fed an HFD, suggesting a phenotype that contrasts with the protective effects observed in females. This difference may reflect sexual dimorphism in metabolic phenotypes, a phenomenon documented in both humans and rodents. For example, women generally exhibit a higher fat composition compared to men [35] and show distinct patterns of fat distribution, favoring subcutaneous fat storage, while men are more prone to visceral fat accumulation [36]. These sex-specific patterns of fat distribution have important metabolic consequences, as visceral adiposity is more strongly associated with cardiometabolic risk. Sexual dimorphism also extends to lipid metabolism, including variations in lipolysis, triglyceride secretion, and clearance [37]. Collectively, these observations indicate that male and female organisms use partially distinct strategies for storing and mobilizing lipids, which likely shape how a given genetic or epigenetic perturbation, such as loss of Dnmt3b in adipocyte progenitor cells, translates into whole-body phenotypes. Mechanistic experiments (e.g., ovariectomy, estrogen receptor blockade, or sex-specific methylome profiling) are required to dissect the basis of this sexual dimorphism.

A well-recognized contributor to these differences is the sex hormone estrogen and its receptors, which play a pivotal role in regulating energy balance, adiposity, and insulin sensitivity. Estrogen signaling has been shown to protect females from HFD-induced weight gain, adipose inflammation, and insulin resistance, in part by promoting subcutaneous rather than visceral fat expansion, enhancing lipid oxidation, and modulating inflammatory pathways in metabolic tissues [38]. Consistent with this, our previous work demonstrated that DNMT1/3A-mediated methylation at the Esr1 promoter in adipocytes suppresses estrogen receptor expression, thereby promoting adipose tissue inflammation and contributing to obesity-induced insulin resistance in female mice [21]. These findings highlight an epigenetic axis linking DNA methyltransferases, estrogen receptor expression, and adipose inflammation. We found in this study that Esr1 expression is increased in all fat depots of female PD3bKO mice, whereas in male PD3bKO mice Esr1 expression is increased in iBAT and shows a trend toward increase in iWAT. Thus, Esr1 expression is elevated in both sexes, but only female PD3bKO mice display a pronounced reduction in adiposity and improved insulin sensitivity. Although the precise mechanism remains unclear, it is plausible that estrogen, acting as the ligand for ERα/ESR1, amplifies the impact of increased Esr1 expression in females. The presence of circulating estrogen in females may synergistically engage upregulated ERα in adipocytes, resulting in a more robust protective metabolic effect compared with males. Although the present study was not designed to dissect these mechanisms directly, our data underscore the importance of considering sex as a biological variable in studies of epigenetic regulation of metabolism and point to future work (e.g., ovariectomy) examining how Dnmt3b-regulated methylation programs interact with estrogen/estrogen receptor signaling in adipose tissue and possibly the central nervous system to generate sex-specific metabolic phenotypes.

In summary, we generated PD3bKO mice with Dnmt3b deficiency in adipocyte progenitor cells and demonstrated that these mice are resistant to HFD-induced obesity and insulin resistance. This resistance is accompanied by enhanced energy expenditure, increased locomotor activity, and reduced caloric intake, which together likely contribute to the observed lean phenotype. Additionally, Dnmt3b deficiency promotes thermogenic programming in brown fat. Interestingly, male PD3bKO mice exhibit no significant changes in body weight but display reduced insulin sensitivity, highlighting a sexually dimorphic metabolic phenotype in this knockout model. Overall, our findings demonstrate that *Dnmt3b* in adipocyte progenitor cells plays a crucial role in regulating energy metabolism and body weight, particularly in female mice.

## 4. Materials and Methods

### 4.1. Mice

Mice with *Dnmt3b* knockout in adipocyte progenitor cells (PD3bKO) were generated by crossing *Dnmt3b*-floxed mice (Mutant Mouse Regional Resource Centers (MMRRC), stock # 029887) with Platelet-derived growth factor receptor alpha (PDGFRα) Cre mice (Jackson Laboratory, Stock # 013148; Bar Harbor, ME, USA). The *Dnmt3b*-floxed mouse was created by inserting two loxP sites flanking exons 16–19, which encodes the catalytic motif [39] and has been backcrossed to B6 background for more than five generations.

### 4.2. Metabolic Analysis

All animal procedures in this study were approved by the Institutional Animal Care and Use Committee at Georgia State University (GSU) (Protocol Number: A19003; Approval Date: 4 September 2018). Mice were housed in a temperature- and humidity-controlled facility at GSU under a 12-h light/dark cycle with free access to food and water. PD3bKO mice and their flox/flox (fl/fl) littermate controls were randomly assigned and fed either a chow diet or a high-fat diet (HFD) (Research Diets D12492, 60% calories from fat) for up to 24 weeks. Identification of the mice was blinded to the researchers who handled the animal study. During the HFD feeding study, body weight was measured weekly, and food intake was monitored in a single cage over seven consecutive days. Body composition, including fat and lean mass, was analyzed using a Minispec NMR body composition analyzer (Bruker BioSpin Corporation, Billerica, MA, USA). Energy expenditure parameters, such as oxygen consumption, carbon dioxide production, and locomotor activity, were measured using the PhenoMaster metabolic cage system (TSE Systems, Chesterfield, MO, USA). Blood glucose levels were measured with a OneTouch Ultra Glucose meter (LifeScan, Milpitas, CA, USA), and glucose tolerance tests (GTT) and insulin tolerance tests (ITT) were conducted to evaluate glucose tolerance and insulin sensitivity, as previously described [32]. At the end of the experiments, various tissues—including all fat pads—were dissected, weighed, and harvested for further analyses, including mRNA expression, protein expression, and immunohistochemistry.

### 4.3. Quantitative RT-PCR

Briefly, total RNA was extracted from fat tissue using the Tri Reagent kit (Molecular Research Center, Cincinnati, OH, USA). mRNA levels of target genes were measured using a one-step quantitative RT-PCR protocol with the TaqMan Universal PCR Master Mix kit (ThermoFisher Scientific, Waltham, MA, USA) on an Applied Biosystems QuantStudio 3 real-time PCR system (ThermoFisher Scientific). TaqMan primers and probes for all target genes were purchased from Applied Biosystems (ThermoFisher Scientific, Waltham, MA, USA).

### 4.4. Histology

Fat tissues were fixed in 10% neutral formalin, embedded in paraffin, and sectioned into 5 µm-thick slices. These sections were processed for hematoxylin and eosin (H&E) staining. Sample identification was double-blinded to examiners for image analysis.

### 4.5. Statistics

We included both male and female mice (8 mice per group), with a sample size based on prior experience and power analysis (SD = 25% of the mean) providing 80% power to detect group differences at α = 0.05. Data were presented as mean ± SEM. Different groups in each experiment were compared for difference by one-way ANOVA or *t* test as appropriate. Statistical significance is accepted at *p* < 0.05.

## Data Availability

The original contributions presented in this study are included in the article/Supplementary Materials. Further inquiries can be directed to the corresponding authors.

## Supplementary Materials

The following supporting information can be downloaded at: https://www.mdpi.com/article/10.3390/ijms27020861/s1.
