# Supplementary material for: Dnmt3b Deficiency in Adipocyte Progenitor Cells Ameliorates Obesity in Female Mice
Source: Int J Mol Sci. 2026 Jan 15;27(2):861. doi: 10.3390/ijms27020861 (PMC12840943; doi:10.3390/ijms27020861)
Supplement: Supplementary file 1 [file ijms-27-00861-s001.zip › ijms-4020252-supplementary.pdf]

*Supplementary File*

# *Dnmt3b* Deficiency in Adipocyte Progenitor Cells Ameliorates Obesity in Female Mice

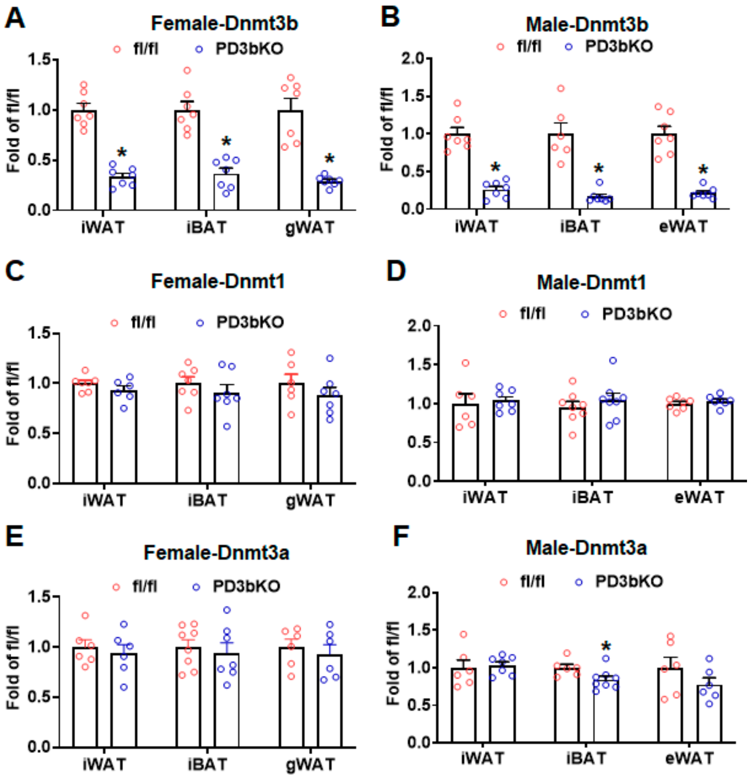


**Figure S1.** (A) Dnmt3b mRNA levels in iWAT, iBAT, and gWAT of female PD3bKO and fl/fl mice (n=6/group). (B) Dnmt3b mRNA levels in iWAT, iBAT, and eWAT of male PD3bKO and fl/fl mice (n=6/group). (C) Dnmt1 mRNA levels in iWAT, iBAT, and gWAT of female PD3bKO and fl/fl mice (n=6/group). (D) Dnmt1 mRNA levels in iWAT, iBAT, and eWAT of male PD3bKO and fl/fl mice (n=6/group). (E) Dnmt3a mRNA levels in iWAT, iBAT, and gWAT of female PD3bKO and fl/fl mice (n=6/group). (F) Dnmt3a mRNA levels in iWAT, iBAT, and eWAT of male PD3bKO and fl/fl mice (n=6/group). All data are expressed as Mean ± SEM; *$p < 0.05$ vs. fl/fl.

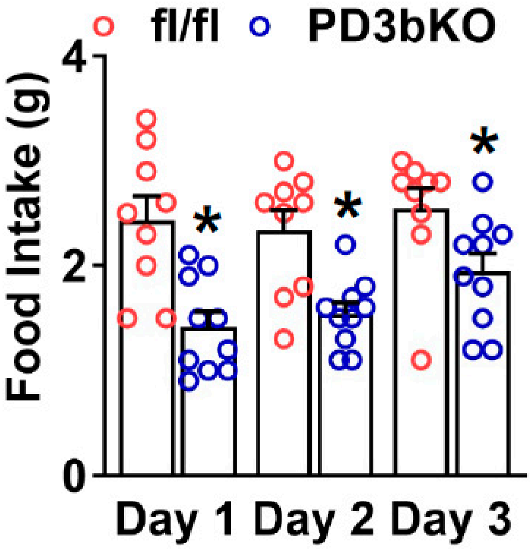


**Figure S2.** Food intake of female PD3bKO and fl/fl mice fed an HFD. All data are expressed as Mean ± SEM; n=9-10/group; *$p < 0.05$ vs. fl/fl.
